# Effects of intravascular photobiomodulation on cognitive impairment and crossed cerebellar diaschisis in patients with traumatic brain injury: a longitudinal study

**DOI:** 10.1007/s10103-023-03764-8

**Published:** 2023-04-20

**Authors:** Yen-Po Lin, Chih-Hung Ku, Cheng-Chiang Chang, Shin-Tsu Chang

**Affiliations:** 1 Department of Medical Education and Research, Kaohsiung Veterans General Hospital, Kaohsiung, Taiwan; 2 Department of Medical Education and Research, National Cheng Kung University Hospital, Tainan, Taiwan; 3 School of Public Health, National Defense Medical Center, Taipei, Taiwan; 4 Department of Physical Medicine and Rehabilitation, School of Medicine, Tri-Service General Hospital, National Defense Medical Center, Neihu District, # 161, Section 6, Minquan East Road, Taipei, 114201 Taiwan; 5 Department of Physical Medicine and Rehabilitation, Kaohsiung Veterans General Hospital, Zuoying Dist., # 386, Dazhong 1st Rd., 813414 Kaohsiung, Taiwan

**Keywords:** Head injury, Cerebellar diaschisis, Rancho Los Amigos Levels of Cognitive Functioning, Intravenous laser irradiation of blood, Single photon emission computed tomography

## Abstract

The association between intravascular photobiomodulation (iPBM) and crossed cerebellar diaschisis (CCD) and cognitive dysfunction in patients with traumatic brain injury (TBI) remains unknown. We postulate that iPBM might enable greater neurologic improvements. The objective of this study was to evaluate the clinical impact of iPBM on the prognosis of patients with TBI. In this longitudinal study, patients who were diagnosed with TBI were recruited. CCD was identified from brain perfusion images when the uptake difference of both cerebella was > 20%. Thus, two groups were identified: CCD( +) and CCD( −). All patients received general traditional physical therapy and three courses of iPBM (helium–neon laser illuminator, 632.8 nm). Treatment assemblies were conducted on weekdays for 2 consecutive weeks as a solitary treatment course. Three courses of iPBM were performed over 2–3 months, with 1–3 weeks of rest between each course. The outcomes were measured using the Rancho Los Amigos Levels of Cognitive Functioning (LCF) tool. The chi-square test was used to compare categorical variables. Generalized estimating equations were used to verify the associations of various effects between the two groups. *p* < 0.05 indicated a statistically significant difference. Thirty patients were included and classified into the CCD( +) and CCD( −) groups (*n* = 15, each group). Statistics showed that before iPBM, CCD in the CCD( +) group was 2.74 (exp 1.0081) times higher than that of CCD( −) group (*p* = 0.1632). After iPBM, the CCD was 0.64 (exp-0.4436) times lower in the CCD( +) group than in the CCD( −) group (*p* < 0.0001). Cognitive assessment revealed that, before iPBM, the CCD( +) group had a non-significantly 0.1030 lower LCF score than that of CCD( −) group (*p* = 0.1632). Similarly, the CCD( +) group had a non-significantly 0.0013 higher score than that of CCD( −) after iPBM treatment (*p* = 0.7041), indicating no significant differences between the CCD( +) or CCD( −) following iPBM and general physical therapy. CCD was less likely to appear in iPBM-treated patients. Additionally, iPBM was not associated with LCF score. Administration of iPBM could be applied in TBI patients to reduce the occurrence of CCD. The study failed to show differences in cognitive function after iPBM, which still serves as an alternative non-pharmacological intervention.

## Introduction

The symptoms of traumatic brain injury (TBI) can range from mild changes in consciousness to severe coma and death. These are affected by diffuse damage and swelling throughout the brain [1]. TBI results in structural damage and produces neurocognitive impairments, inducing to impairments in attention, memory, and executive function [2]. Cognitive deficits may appear in patients with TBI in the first few hours [3–5] after trauma; they can affect patients’ ability to perform certain tasks up to 3–5 days after injury, such as immediate recall, short- or long-delay recall, attention, work, processing speed, memory, and other executive functions [6–8]. Therefore, a broad range of cognitive factors have been investigated as predictors of outcome in patients with TBI [9].

Moreover, some symptoms, including ataxia, postural instability, tremor, and impairments in balance and fine motor skills, are found in TBI. These may be attributed in part to cerebellar damage or so-called hypometabolism [10] due to the functional separation of the contralateral hemisphere from the cerebral cortex. This was first described clinically in 1914 as contralateral symptoms in the cerebral and cerebellar hemispheres [11, 12].The underlying mechanism may be a loss of afferent stimuli due to damage of the cerebro-ponto-cerebellar pathways, which were first discovered in humans in 1980 [13, 14]. Research using single-photon emission computed tomography (SPECT) and positron emission tomography have shown a diminution of blood flow and hypo-metabolism in the cerebellar hemisphere contralateral to the supratentorial region. This was defined as crossed cerebellar diaschisis (CCD) [15]. Similar lesions include cerebral hematoma, head injury, and some kind of epilepsy. The presence of CCD in TBI has been associated with an unfavourable clinical outcome [16, 17]. The persistence of CCD could result in atrophy in the affected cerebellar hemisphere [11, 18]; CCD has been related to poor neurological improvements in the affected cerebellum after TBI [16, 18]. Therefore, it is important to find appropriate treatments that improve cerebellar function, correlated with hypoperfusion volume.

Photobiomodulation (PBM) is basically a function of energy absorption by utilizing non-ionizing forms of light sources to induce cascade of biological reactions in a specific photoacceptor [19]. PMB can be accomplished using lasers, nightline-emitting diodes (LEDs), and other light sources, usually at wavelengths in 400–1100 nm [20]. Both LED and laser sources are characterized using the same methods [21, 22]. PMB methods are currently used in clinical practice with demonstrated safety and efficacy for various different indications, for example, cartilage/bone defects [23, 24], pain [25], wound healing [26], and a wide range of neurological disorders [27].

Intravascular photobiomodulation (iPBM) has been advocated as a non‐pharmacological treatment, and new articles sprang up like bamboo shoots after a spring rain since 1990. The use of iPBM shows high efficacy in the treatment of diabetes, coronary artery disease, and other pathological processes over the past few decades [28–31]. To date, iPBM can improve sleep disturbances and has been applied in ischemic stroke in humans [32–36]. However, to the best of our knowledge, no study has explored the association between iPBM and CCD in patients with TBI. Both CCD and cerebral ischemic stroke are low flow states and we have shown good functional recovery when administering iPBM in CCD in a previous case report [34]; therefore, iPBM may be an effective symptomatic treatment.

We postulate that this intervention may enable better neurologic improvements in patients with TBI. Our study employed a longitudinal method to confirm the effect of iPBM on individuals with TBI. Rancho Los Amigos Levels of Cognitive Functioning (LCF) and SPECT measurements were used to assess the cognitive function and CCD conditions before and after the improvement from iPBM, respectively.

## Materials and methods

### Patients

**Fig. 1:**
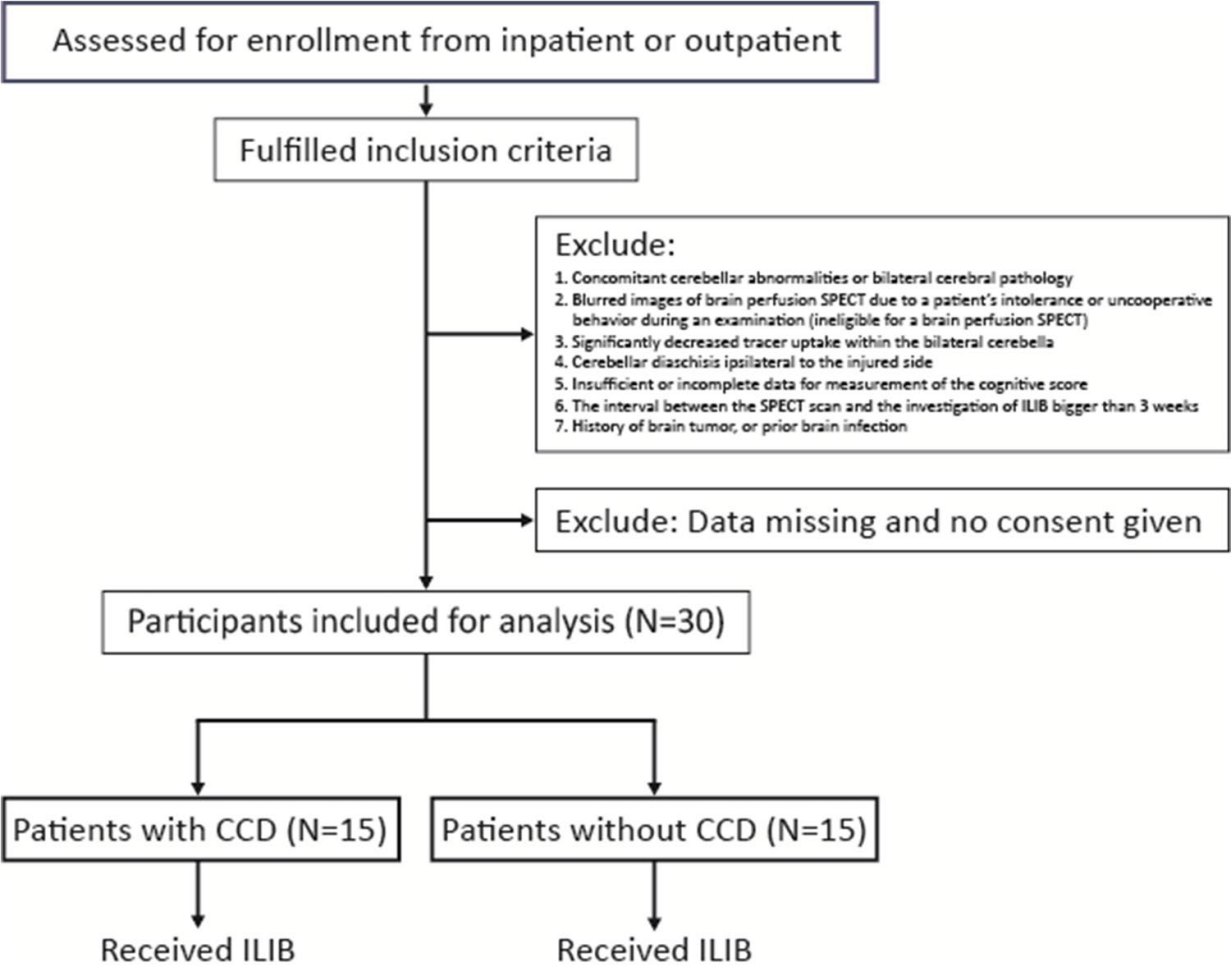
Flowchart of the study

The human ethics committee of the medical centre approved this study (TSGHIRB No. 2–101-05–049), which conformed to the principles of the Declaration of Helsinki. This was a prospective study. Patients who were diagnosed with TBI were recruited from the inpatient or outpatient areas of the Tri-Service General Hospital, Taiwan (Fig. 1).

Inclusion criteria were as follows: (1) history of traumatic brain injury in the recent 3 years without or with surgical intervention, including ventriculoperitoneal shunting; (2) regional brain perfusion SPECT conducted in the Department of Nuclear Medicine; and (3) mild to moderate cognitive impairment as measured by the LCF.

Exclusion criteria were (1) simultaneous cerebellar abnormalities or cerebral pathology in both sides; (2) blurred brain perfusion SPECT images owing to intolerance or uncooperative behaviour of patient during test (disallowed for an brain perfusion SPECT); (3) significantly reduced tracer uptake within the both cerebella; (4) cerebellar diaschisis ipsilateral to the injured side; (5) insufficient or incomplete LCF data measurements; (6) the interval between the SPECT scan and investigation of iPBM was longer than 3 weeks; and (7) a history of brain tumour, or prior brain infection.

### Examination of SPECT images

Brain SPECT images were acquired after intravenous shot of the tracer, ethyl cysteinate dimer (Tc-99 m-ECD). This was purchased from a commercial kit (Neurolite Du Pont Merck Pharmaceutical Company, Billerica, MA, USA) and prepared by including 25 mCi of freshly eluted Tc-99 m pertechnetate to 5 mL of saline solution. Imaging commenced 30 min after injection of the radiotracer using a dual-headed camera furnished with very high-resolution fan-beam collimators. Data were obtained in a 128 × 128 matrix with a 1.4 × zoom through 360° (180° for each head) with rotation at 3° intervals, for 30 s per angle step. Images were built utilizing a back-projection method [37] with a Metz filter. Images of SPECT were analysed from three-dimensional space (axial, sagittal, and coronal slices, with a 0.3-cm slice thickness) and earned a full set of axial tomographic slices from the posterior cranial fossa to the vertex. CCD was determined while the uptake difference between cerebellar hemispheres was > 20% [38, 39]. No CCD was identified as a < 20% difference in uptake between cerebellar hemispheres and between the left (or right) cerebellum and the adjacent occipital lobe on the SPECT images. Accordingly, two groups were classified as CCD( +) and CCD( −).

### Outcome measurements

Cognitive evaluation was performed using the LCF [40] tool before and after iPBM in the neurorehabilitation unit by a skilled neurophysiatrist. Investigators extracted essential clinical characteristics from the electronic medical records, for instance, demographics, injury type, days from injury to unit admission, length of stay, and diagnostic imaging.

### Intervention of iPBM

Patients with CCD received three courses of iPBM (Taiex He–Ne Laser, YJ-ILIB-5, Bio-ILIB Human Energy Corporation, Taiwan). This was applied with visible red light at a wavelength of 632.8 nm, power output 2.5–3.8 mW (milliwatts), power intensity 1.28–1.94 W/cm^2^, total energy 9.00–13.68 J, exposure time 60 min, and energy density 4591.84–6976.60 J/cm^2^ [41]. This was commenced at an easily reached peripheral vein via an optical fibre 0.5 mm in diameter through a phlebotomy cannula. Laser power was adjusted depending on the patient’s sleeping responses. The initial output dosage for shooting adopted the ratio measured as body mass index (BMI) divided by 10; i.e., at BMI 25, the dosage is 2.5 mW, and then the shooting dosage is increased by 0.1 mW per day. When the patient reports that the sleep night before was a bit tossed and turned, or he/she is too exciting to fall asleep, it means that the dosage has reached the highest bearing point to the patient. We therefore lower down to yesterday’s value, and then maintained it until the end of the treatment.

The sum of blood irradiated in an assembly was hypothesized to be near 100% of the total blood volume, as the regional-arm-to-brain mean transit time calculated using a radionuclide required < 30 s [42]. Based on this fact, the cycle time of blood passed through the circulatory system would not be more than 1 h. Hence, all the blood was most likely irradiated by the laser light during each 60-min session. We slowly but surely raised the power output from 2.5 to 3.8mW as the assemblies progressed accountable on patients’ responses. Treatment assemblies were performed on weekdays for 2 consecutive weeks as a solitary treatment course; 3 treatment courses were performed over 2–3 months, with 1–3 weeks of rest between each course [43].

### Statistics

The differences in characteristics of those patients were compared between the two groups (*n* = 15 per group). The chi-square test was used for categorical variables. Generalized estimating equations (GEEs) were used to verify the associations of varying effects (group, age, and handedness) in the two groups and to adjust the potential confounders (age and interval duration). A *p* value of 0.05 or less indicated a statistically significant difference. After establishing the concluding GEE models for CCD and LCF score, contrast estimates were then calculated. All data analyses were carried out using the PROC GENMOD function of SAS version 9.4 (Carry, NC, USA).

## Results

Thirty patients with a diagnosis of mild-to-moderate TBI that fit the inclusion criteria completed clinical testing and treatment (*n* = 15 patients per group). The interval between TBI onset and the time of the iPBM ranged from 0.7 to 1.8 years. The interval between the SPECT scan and the intervention of iPBM ranged from 3 to 7 days. Table 1 presents the baseline demographic characteristics of the study cohort. The eligible sample was composed of 25 men and 5 women, age 26–56 years (Table 1). Twenty-nine patients were right-handed and 1 was left-handed. There were no significant differences in the demographic characteristics between groups.

**Table 1:** Demographic characteristics

	CCD( +) group	CCD( −) group	*p* value
Male sex—no. (%)	12 (80.0)	13 (86.7)	0.19
Mean (± SD) age at TBI onset—year	41.2 ± 18.4	42.1 ± 20.3	0.08
Head surgery for TBI—no. (%)	8 (53.3)	9 (60.0)	0.12
Side of hemiplegia/hemiparesis—no. (%)			0.35
Left	8 (53.3)	7 (46.7)	
Right	7 (46.7)	8 (53.3)	
Handedness—no. (%)	0:15	1:14	0.43
Left	0	1 (6.7)	
Right	15 (100)	14 (93.3)	
TBI severity (based on LCF scale)—no. (%)			0.23
Level 5	1 (6.7)	0	
Level 6	6 (40.0)	8 (53.3)	
Level 7	8 (53.3)	7 (46.7)	
Coexisting conditions—no. (%)			
Cardiovascular disease	4 (26.7)	3 (20.0)	0.68
Hypertension	6 (40.0)	8 (53.3)	0.59
Type 2 diabetes mellitus	2 (13.3)	3 (20.0)	0.81

For the LCF scale, level V indicates a confused, non-agitated state with inappropriate behaviours, and needing maximal assistance; level VI indicates a confused state, with appropriate behaviours, the ability to follow simple commands, and needing moderate assistance; and level VII indicates automatic and appropriate behaviours and needing minimal assistance for daily living skills

*SD* standard deviation, *TBI* traumatic brain injury, *LCF* Rancho Los Amigos Levels of Cognitive Functioning

After GEEs accounting for between-group difference, within-group variation, and the group-by-time interaction effects, we found that before iPBM, the CCD( +) group had a 2.74 (exp 1.0081) times higher CCD than the CCD( −) group (Table 2, *p* = 0.1632). After iPBM, the CCD( +) had a 0.64 (exp − 0.4436) times lower CCD than the CCD( −) group (Table 2, *p* < 0.0001). Cognitive assessment revealed that, before iPBM, the CCD( +) group had a non-significantly 0.1030 lower LCF score than that of CCD( −) group (Table 3, *p* = 0.1632). Similarly, the CCD( +) group had a non-significantly 0.0013 higher score than that of CCD( −) after iPBM treatment (Table 3, *p* = 0.7041), indicating no significant differences between the CCD( +) or CCD( −) following iPBM and general physical therapy.

**Table 2:** Contrast estimate for GEE model for occurrence of CCD between CCD( +) group and CCD( −) group

Empirical standard error estimates							
Parameter			Estimate ± SE	95% confidence limits		*Z*	*p*-value
Intercept			− 1.3863 ± 0.6455	− 2.6514	− 0.1211	− 2.15	0.0317
Group	1		1.0081 ± 0.7229	− 0.4087	2.4249	1.39	0.1632
time	1		− 0.0000 ± 0.0000	− 0.0000	0.0000	− 0.00	1.0000
Group × time	1	1	− 0.4436 ± 0.1017	− 0.6429	− 0.2443	− 4.36	< .0001
Contrast estimate results							
Label			L’Beta	L’Beta		Chi-square	Pr > ChiSq
			Estimate ± SE	95% confidence limits			
CCD( +) vs. CCD( −) at time 0			1.0081 ± 0.7229	− 0.4087	2.4249	1.94	0.1632
ExpCCD (+ vs. −) at time 0			2.7403 ± 1.9809	0.6645	11.3012		
CCD( +) vs. CCD( −) at time 1			− 0.4436 ± 0.1017	− 0.6429	− 0.2443	19.03	< .0001
ExpCCD (+ vs. −) at time 1			0.6417 ± 0.0653	0.5258	0.7833		

*SE* standard error, *CCD* crossed cerebellar diaschisis

**Table 3:** Contrast estimate for GEE Model for Rancho Los Amigo Cognitive Score between the CCD( +) group and the CCD( −) group

Empirical standard error estimates							
Parameter			Estimate ± SE	95% confidence limits		*Z*	*p*-value
Intercept			6.0849 ± 0.1954	5.7020	6.4678	31.15	< .0001
Group	1		− 0.1030 ± 0.3252	− 0.7404	0.5344	− 0.32	0.7514
time	0		− 0.0079 ± 0.0025	− 0.0127	− 0.0030	− 3.16	0.0016
Group × time	1	0	0.0013 ± 0.0034	− 0.0054	0.0081	0.38	0.7041
Contrast estimate results							
Label			L’Beta			Chi-square	Pr > ChiSq
			Estimate ± SE	95% confidence limits			
CCD( +) vs. CCD( −) at time 0			− 0.1030 ± 0.3252	− 0.7404	0.5344	0.10	0.7514
CCD( +) vs. CCD( −) at time 1			0.0013 ± 0.0034	− 0.0054	0.0081	0.14	0.7041

*SE* standard error, *CCD* crossed cerebellar diaschisis

## Discussion

This study assessed the clinical impact of iPBM on the prognosis of patients with TBI with CCD( +) or CCD( −). We found that the phenomenon of CCD could be reduced in iPBM-treated patients. Furthermore, we found that the iPBM was not associated with LCF score. To the best of our knowledge, this is the first study to assess the associations between iPBM treatment and the outcomes of the patients with TBI.

Poor long-term outcomes in TBI, including headache, cognitive difficulties, and imbalance, may frequently co-exist with CCD [44]; therefore, long-term follow-up and specific interventions are important in patients with TBI [45]. Several studies have investigated the effect of specific interventions, such as iPBM on ischemia stroke; however, the consequences of these treatments on the occurrence of CCD in TBI remain unclear. In the present study, we found that patients using iPBM effectively reduced the incidence of CCD. This effect was not associated with any traditional physical or occupational therapies. Previous animal experiments have indicated that ordinary PBM may induce neurogenesis, thus providing a significant functional benefit [46].

The efficacy of laser therapy may be multifaceted. First, iPBM promotes functional regeneration and alleviates oxidative stress and mitochondrial dysfunction in living bodies [43]. This has been shown in patients with spinal cord injury (SCI) via the stimulation of enhanced ATP synthesis, which contributes to the clear signalling of free radicals [41, 47]. SCI and TBI co-occur frequently; similar neurorehabilitation has been shown to improve motor function in SCI and TBI, and both share identical mechanism for neurological recovery [48, 49]. A common secondary injury of TBI is from mitochondrial damage dysfunction. This directs to oxidative stress, reduced cellular energy production, and possible apoptosis [50]. Therefore, we hypothesize that our findings may be partly attributable to the positive effects of photo stimulation on mitochondrial proliferation and/or cellular homeostasis. Secondly, CCD is a negative prognostic factor after stroke or TBI for the reason that it is related to regional hypoperfusion in the cerebellar hemisphere. In our previous study, iPBM promoted perfusion in stroke patients with CCD [24]. However, other study in patients with brain injury and CCD showed no effective treatment could improve and/or eradicate CCD. Therefore, we hypothesize that patients with TBI and CCD should receive benefits from iPBM treatment with similar efficacy to patients following stroke. We used SPECT based on objective data with cerebral perfusion imaging to measure changes in brain and cerebellum perfusion after iPBM. This is more relevant than making a clinical diagnosis. Our study confirmed that iPBM can lead to a significant promotion in cerebellar perfusion, thereby reducing CCD. Further experimental studies are needed to explore the underlying mechanisms of CCD elimination in patients following iPBM treatment.

We evaluated cognitive function using the Rancho Los Amigos LCF tool. We observed no significant differences between the patients following iPBM and general physical therapy. The possible explanation is that LCF could not correspond with the dimensional structure of the original tool [51]. Currently, a variety of physical therapy approaches, such as mind–body exercises, resistance, and strength training, has been shown to help improve cognitive function [52, 53]. This is significant due to the influence of TBI onset-test interval increases. Our results do not support the theory that iPBM therapy can achieve similar improvements in cognitive function as general physical therapy, even laser biostimulation has been confirmed as a valuable tool in preventing or delaying age-related cognitive decline [54]. A possible reason for this conflict is that continuous and adequate cerebral blood flow is essential for neurological function, and its reduction exacerbates cognitive ability [55, 56], but not feasible in all situations. Accordingly, these symptoms can be alleviated by the perfusion effect of iPBM treatment. A recent review has reported that transcranial near-infrared laser is a promising manner to improve cognitive impairments following TBI [57]. While this intervention differs from ours, it does support the value of improving cognitive function in patients with TBI via non-pharmacological or surgical treatments. The use of iPBM has high accessibility and few contraindications. Its benefit is important for Taiwanese people compared to other countries because they are allowed to freely choose their clinic and medical facilities without referral [58]. Taken together, we postulate that iPBM could enhance cognitive function like other physical therapies; therefore, it serves as an alternative treatment option. However, additional studies are needed to confirm this hypothesis.

With respect to the matter of ipsilateral cerebellar diaschisis (ICD), one of exclusion criteria, only very few articles have been documented as opposed to CCD. Lenzi et al. first reported three cases of 15 adults with cerebral ischemia having ICD [59]. ICD also occurs in patients with intraparenchymal hematoma after severe head injury [60]. The infarcts of medulla and pons, particularly the lesions of some fibers like caudal or lateral tissue that comprise middle cerebellar peduncle, will result in ICD [61, 62]. ICD was first reported in children before the age of 6, who had acute subdural hematoma or acute hemiplegia in the infant or perinatal stage, and its pathophysiology is assumed to be differences in maturation of the cortico-ponto-cerebellar fibers during childhood [61, 63]. There are two paths linking the cerebral cortex together the cerebellum, the cortico-ponto-cerebellar and cortico-olivo-cerebellar tracts, and both are predominantly crossed. On the other hand, the spino-olivary tract is both crossed and uncrossed [64], and ICD might be attributed to the restraint of the uncrossed part of the tract. In addition, Kernohan-Woltman notch phenomenon as another mechanism to cause ICD cannot be ignored. The exact mechanism for ICD needs further investigation, and its prognostic nature is not fully understood. That is the reason we listed ICD as one of exclusion criteria in this study.

There are some potential limitations to our study. First, the study design collected data for only 3 months; therefore, we could not assess any long-term effects. Second, the sample size was small (*n* = 30). Even so, we add the pre- and post-iPBM material up to obtain 60 sets of data, which is still an opportunity to implement statistics. Further, we used the GEEs, which itself is designed to target small sample size, and yielded meaningful results in CCD times post-treatment. Finally, our study did not assess the effects of different wavelengths, duration, dose, energy density, and power density for each individual treatment. In addition, multiple iPBMs were performed; further studies are needed to clarify the duration until tolerance develops.

## Conclusion

CCD is a negative prognostic factor for neurological recovery. Administration of iPBM could be applied in patients with TBI patients to reduce the occurrence of CCD. The study failed to show differences in cognitive function after iPBM. Therefore, it serves as an alternative non-pharmacological and surgical intervention.

## Declarations

The human ethics committee of the medical centre approved this study (TSGHIRB No. 2–101-05–049), which conformed to the principles of the Declaration of Helsinki. With the approval of the Institutional Review Board, informed consent was also obtained from all participants.
